# Adherence to the Revised National Malaria Case Management Guidelines Among Healthcare Workers at Mzimba District Hospital in Northern Malawi

**DOI:** 10.1002/puh2.70371

**Published:** 2026-10-08

**Authors:** Rita Luhanga Soko, Chisomo Mulenga, Thokozani Mzumara, Mandayachepa Clifford Nyando

**Affiliations:** ^1^ Mzuzu Urban Community Hospital Mzimba North District Health Office Mzuzu Malawi; ^2^ Kamuzu University of Health Sciences Lilongwe Malawi

**Keywords:** adherence, child health, quality, revised national malaria guidelines, severe malaria

## Abstract

**Background:**

Management of severe malaria among children remains a challenge in sub‐Saharan Africa, including Malawi. Therefore, this study examined the adherence to the national malaria case management guidelines among Malawian healthcare workers.

**Methods:**

This was a mixed‐methods study that used purposive sampling to recruit nurses and clinicians in paediatric and nursery wards. Quantitative data were collected from 33 participants through a clinical observational checklist. Qualitative data were collected from 12 participants through in‐depth interviews. Quantitative data were analysed using SPSS version 22.0, and qualitative data were analysed using thematic analysis.

**Results:**

The results indicated that 32/33 of the participants effectively treated under‐five children with severe malaria, whereas 23/33 of the participants made incorrect diagnoses. Additionally, the general paediatric ward nurses did not appropriately monitor the patients. Most healthcare workers failed to provide adequate health education and follow‐up appointments for the discharged children and their guardians due to a shortage of staff, lack of equipment and lack of commitment.

**Conclusion:**

The study suggests that there is poor adherence to the national malaria guidelines on the management of children with severe malaria.

## Graphical abstract

The study suggests that there is poor adherence to the national malaria guidelines as evidenced by incorrect diagnoses, poor monitor of patients, lack of adequate health education and follow‐up appointments for the discharged children and their guardians due to a shortage of staff, lack of equipment and lack of commitment.

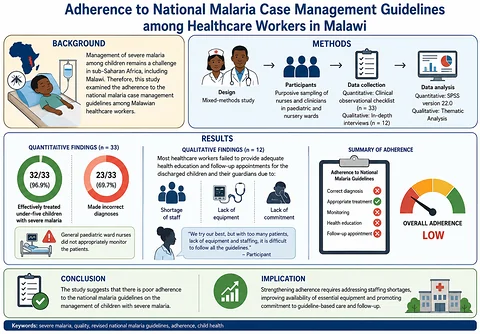

## 1 Introduction

Malaria remains one of the high‐burden diseases that distresses public health and development in the world, especially in sub‐Saharan Africa (SSA), including Malawi [1]. Despite recent efforts to curb malaria, it remains one of the leading causes of mortality particularly among children, due to their insufficient acquired immunity [2]. The World Health Organisation (WHO) [3] report points out concerning trends in malaria deaths, indicating an increase from 13 per 100,000 population at risk in 2019 to 15 per 100,000 deaths in 2020, with a higher number of deaths among children under 5 years old globally. The African region accounted for a substantial portion, contributing to 95% and 96% of the world's malaria cases and deaths, respectively. This shows that despite the implementation of various malaria prevention strategies, such as case management, insecticide‐treated nets, paediatric malaria management, procurement and supply chain, as well as behaviour change communication, malaria‐related deaths still occur in children below 5 years [4].

Malawi recorded an estimated 6.9 million malaria deaths in 2021, and 9% of these were among children. To ensure effective case management, the Malawi National Malaria Control Programme (NMCP) revised the guidelines for managing malaria in 2020 to align with the current evidence by WHO [5]. The WHO defines severe malaria as a medical emergency that requires parenteral treatment as it may result in death in 24 hours [6]. According to the Malawi revised guideline for the management of malaria, severe malaria is defined by the presence of one or more severe clinical or laboratory findings [7]. Though the guidelines emphasise prompt diagnosis following correct treatment similar to many low‐ and middle‐income countries (LMICs), the implementation of malaria guidelines in Malawi remains sketchy [8].

Accordingly, it is essential to monitor patients effectively; however, evidence indicates that hospitalised patients with severe malaria are not properly monitored due to a lack of training on malaria guidelines, inadequate equipment and a shortage of staff [9]. Consistent monitoring of hospitalised children can lead to a good prognosis [10]. Therefore, the admission process and inpatient care practices, such as monitoring, should be investigated [11]. Furthermore, the protocol recommends the provision of health education to prevent the recurrence of the condition and the provision of follow‐up dates to monitor late complications [12]. Irrespective of the guidelines, only a few patients receive education with no or little information on follow‐up visits [13]. Another important aspect is regarding patient follow‐up [14]. Regardless of the needs for competent health providers to effectively manage and implement guidelines, children below 5 years continue dying from malaria infection due to compromised management [15].

Research conducted in Kenya has demonstrated that knowledge of the treatment of severe malaria among healthcare workers is lacking [16]. On the contrary, a study conducted over a decade ago indicated that Malawian doctors and pharmacists have knowledge regarding malaria treatment guidelines [17]. Another study conducted in Malawi found that there were inconsistencies in the diagnosis and documentation of severe malaria, but patients with malaria were given the recommended treatment [7]. Clearly, the literature depicts conflicting evidence regarding the adherence of healthcare workers to the malaria treatment guidelines, depending on study population, health system variations and socio‐political context.

Nevertheless, the healthcare workers’ adherence to treatment guidelines for severe malaria among children in Malawi remains unknown. Since severe malaria is a major cause of preventable childhood death in countries such as Malawi [18], the study bridges this gap by examining the adherence to the revised national malaria case management guidelines among healthcare workers attending the management of under‐five children with severe malaria. Accordingly, strict compliance with the recommended treatment guideline is integral to the effective management of malaria and reduction of child mortality [19]. Hence, the findings of this study will aid in the management of severe malaria by informing interventions aimed at improving the quality of care and reducing child mortality due to malaria.

## 2 Methodology

### 2.1 Study Design and Setting

This was a mixed‐method cross‐sectional study conducted at the nursery and general paediatric wards of Mzimba District Hospital in Northern Malawi. Malawi is a landlocked country in southeast Africa surrounded by Tanzania, Zambia and Mozambique [20].

#### 2.1.1 Quantitative: Population, Sampling and Sample Size

The study used census sampling to select all nurses and clinicians working in both paediatric and nursery wards. As such, 33 participants were recruited.

#### 2.1.2 Quantitative Data Collection Method and Instrument

Quantitative data were collected through an observational checklist adapted and modified from the supervisory checklists used in the Malawi NMCP and Service Provision Assessment (SPA) survey. The checklist aims to ensure quality of care, proper diagnosis and adherence to national malaria guidelines [21]. Data collection was done by the PI, who is a qualified nurse with extensive experience in conducting malaria supervision programmes.

### 2.2 Quantitative Data Analysis

After data collection, the checklists were checked for completeness, entered, cleaned and processed using SPSS version 22.0. Descriptive statistics were presented using frequencies and percentages, and we used tables, charts and graphs to illustrate the data graphically. Furthermore, we used the chi‐square test to assess the association between variables. The value of *p* < 0.05 was considered statistically significant.

#### 2.2.1 Qualitative: Population, Sample Size and Sampling Technique

A qualitative sample was drawn from the same nurses and clinicians working in both paediatric and nursery wards, and participants were selected purposefully. On the basis of data saturation, the study included 12 participants using convenience sampling.

#### 2.2.2 Qualitative Data Collection

A semi‐structured interview guide adapted and modified from the SPA and NMCP supervisory tools was used to collect data and was presented in English. During participant recruitment, the researcher sought consent by reading and explaining all information on the information sheet about the study to allow participants to make an informed decision. The participants who understood the study information and provided written consent form voluntarily before data collection. All of the interviews were recorded in audio format, transcribed word for word, verified for accuracy and summarised with brief notes by the first author. Each interview lasted approximately 30 min.

#### 2.2.3 Qualitative Data Analysis and Management

Thematic analysis was used to analyse the data. The first author prepared the data by transcribing it and organising it while familiarising herself with the data collected by listening to the recordings several times while following the written transcript to avoid errors, omissions and inaccuracies. The data were analysed using thematic analysis. Afterwards, data were categorised and further coded into themes and then sub‐themes. To ensure trustworthiness of the data, we used member checking to allow participants to go through the data and avoid deception. The study used deductive coding to establish the themes a priori based on the research question.

#### 2.2.4 Ethical Considerations

The study was approved by the Kamuzu University of Health Sciences’ College of Medicine Research and Ethics Committee (P/11/22/3855), and permission was sought from the M'mbelwa Director of Health and Social Services through the Mzimba District Hospital research committee. We obtained written informed consent from all the participants before the interview. The quantitative data collection ensured that all participants were kept anonymous through the use of codes by applying the pseudonymisation technique.

## 3 Results

### 3.1 Quantitative Results

#### 3.1.1 Demographic Characteristics

Table 1 shows that according to cadre, the majority of participants were nurses, 12/33, followed by medical assistants, 11/33.

**TABLE 1:** Professional cadre.

Variable	Frequency	Percentages
Medical assistants	11	33.3
Nurse	12	36.4
Clinical officer	10	30.3

The results show that the majority of participants (17/33) inaccurately diagnosed severe malaria, as indicated in Table 2.

**TABLE 2:** Adherence to diagnostic and treatment guidelines.

Variable	Frequency	Percentages
**Diagnosis**		
Correct diagnosis	16	48.5
Incorrect diagnosis	17	51.5
**Treatment**		
Artesunate	33	100

Considering monitoring of under‐five children with severe malaria, 12/33 of healthcare workers checked for vital signs, 10/33 checked for the occurrence of convulsion and only 2/23 checked for parasite density (Table 5). With respect to health education and follow‐up advice provided during discharge, 26/23 were taught how to take medications, whereas 3/33 were taught about drug compliance. However, none were told about the danger signs of malaria, malaria prevention and where to report for follow‐up visits (Table 6). As shown in Table 4, all 27 had a secure IV line, employed 10 mL syringes, had the correct dose from the vial and infused the drug slowly for 20 s. Nevertheless, none of the healthcare workers cleaned the top of the vial with an alcohol swab, whereas 21 pushed the normal saline to check the patency, and only 4 washed their hands after removing gloves.

Additionally, not all the participants followed all the required steps while preparing and administering parenteral artesunate as presented in Tables 3 and 4.

**TABLE 3:** Steps of artesunate preparation (*n* = 27).

Steps	*N*	Frequency	Percentages
Recheck calculation	27	27	100
Lines vials before reconstitution	27	0	0
Clean the top vial with an alcohol swab	27	0	0
Breaks ampoules with a swab	27	0	0
Draw sodium bicarbonate with a sterile syringe	27	27	100
Withdraw air from the vial to create space	27	0	0
Add 5 mL saline to 1 mL of sodium bicarbonate solution	27	27	100
Wait for the contents to clear	27	27	100

**TABLE 4:** Steps in artesunate administration (*n* = 27).

Steps	*N*	Frequency	Percentage
Secure IV line	27	27	100
Use 10 mL syringes	27	27	100
Pushes normal saline to check patency	27	21	77.7
Clean the top of the vial with an alcohol swab	27	0	0
Draw the correct dose from the vial	27	27	100
Infuse the drug slowly for 20 s per dose	27	27	100
Cleans vein with 3 mL of normal saline	27	0	0
Documents on treatment chart time and amount	27	27	100
Discards vial with remaining reconstituted artesunate solution	27	27	100
Remove gloves and wash hands	27	4	14.8

Table 5 summarises the results of the most monitored parameters in the under‐five children with severe malaria. The findings revealed that discharged patients and their guardians did not encounter health education, as required by the guidelines, as shown in Table 6.

**TABLE 5:** Monitoring of under‐five children with severe malaria.

Variable	Frequency	Percentages
Vital signs	12	41.4
Occurrence of convulsion	10	34.5
Ability to feed	9	31
Parasite density	2	6.9

**TABLE 6:** Health education and follow‐up dates provided to discharged under‐five children and their guardians under‐five children with severe malaria (*n* = 27).

Health education message	Frequency	Percentages
How to take drugs	26	96.3
Drug compliance	3	11.1
Danger signs	0	0
Malaria prevention	0	0
When to report for follow‐up	0	0
Schedules for follow‐up up caregiver asked if any questions	0	0

### 3.2 Qualitative Findings

Two major themes emerged from the study: the diagnosis of malaria and interventions for severe malaria. From these, six sub‐ themes emerged. Theme 1 included symptomatic diagnosis and objective diagnosis. Theme 2 included the type of treatment for severe malaria, monitoring of severe malaria, provision of health education and provision of follow‐up data.

#### 3.2.1 Diagnosis of Severe Malaria

##### 3.2.1.1 Symptomatic Diagnosis

The majority of the participants knew the type of history to ask mothers and the physical examination to perform on suspected severe malaria patients. One healthcare worker explained

From history, the guardian will tell you presenting signs like persistent vomiting, fevers, multiple convulsions before coming to the hospital, yellowish eyes, dark coloured urine, and painful urination … poor appetite, and low level of consciousness. On examination … is prostrated, anaemic, convulsing, in a coma, and hypoglycaemic (p. 8).

Another healthcare added

The mother will tell you the child is hot. So we take it as the history of fever, poor appetite, and also some of them just come with low sugars and low level of consciousness (p. 18).

Firstly, you can use clinical judgement by just looking at the child's danger signs like cyanosis, prostration, respiratory distress, and convulsions (p. 3).

##### 3.2.1.2 Objective Diagnosis

All the participants indicated that blood film (Bf) was a gold standard test to diagnose severe malaria. However, some participants stated that both mRDT and Bf are used, plus clinical judgement. The clinical judgement of the clinician based on knowledge and experience is used to select which objective test to run in order to diagnose severe malaria besides the symptoms and signs observed.

Secondly, you can use laboratory investigations we start with mRDT; if the result is positive, we collect the sample for Bf to find out the density of the parasites; we do both due to lack of trust and missing samples (p. 3).

#### 3.2.2 Interventions

##### 3.2.2.1 Type of Treatment for Severe Malaria

The majority of the participants treated severe malaria with artesunate concurrently with antibiotics. A minority of the participants were not prescribing both artesunate and antibiotics to severe malaria patients:

We give antibiotics as routine … we don't have guidelines at hand, hence clinicians prescribe antibiotics for each patient to treat bacterial infections (p. 8).

##### 3.2.2.2 Monitoring of Severe Malaria

All participants mentioned that only clinicians were monitoring patients in the ward and mentioned the level of consciousness, random blood sugar, Bf, vital signs, oxygen saturation, renal function and feeding habits as commonly monitored parameters:

In HDU, monitoring is done every 4 h … in the general ward whenever necessary for very sick patients, it's twice, in the morning during morning ward rounds. Helps to detect danger signs or worsening of disease (p. 16).

We do Bf every 12 h, but here we have the problem with the laboratory guys. Most of the time, even if you get the Bf samples, you go there to follow up on the results, they tell you the samples have been lost. But they are supposed to be monitored 12 hourly (p. 13).

##### 3.2.2.3 Provision of Health Education

The majority of the participants knew the health education messages to give to discharged patients and their guardians, such as information on danger signs, when to come back, drug compliance, how to take drugs at home, sleeping under a mosquito net and cutting tall grass short:

On discharge, health education is given to the mothers on drug compliance and how she will give the medications at home. We also give health education on the importance of sleeping under Long Lasting Insecticide‐treated Net at home and cutting tall glass short (p. 3).

At home, we tell them to observe for danger signs like fever, convulsions, and pallor. We teach them how to take care of the child at home (p. 1).

##### 3.2.2.4 Follow‐Up Dates

Almost all the participants indicated that only special cases are given follow‐up dates for review:

We don't give follow‐up dates to all patients; we give to the patient who needs follow‐up to detect post‐discharge complications. Other patients, we just discharge them without a review date (p. 13).

Others we tell them to come back when they see danger signs at home.

## 4 Discussion

The study examined the processes followed in the management of children with severe malaria based on the revised national malaria guidelines among the nurses and clinicians at Mzimba District Hospital. The quantitative part indicated that the majority of healthcare workers are not adhering to the malaria guidelines. The qualitative findings revealed that the participants lack trust in the guidelines due to health system challenges, such as lost samples.

The findings revealed that the majority of the nurses and clinicians order both mRDT and microscopy at once, citing mistrust and lost samples, contrary to the malaria diagnostic guideline, which recommends diagnosis of severe malaria with Bf only. Somehow this could explain the large number of misdiagnoses of malaria despite healthcare workers’ awareness of symptoms of severe malaria. The results were in line with studies in Sudan, Ghana, Uganda and Malawi, where the majority of the patients with severe malaria were diagnosed with both mRDT and Bf at once, with none of the patients being checked for Bf due to the lack of trust in the laboratory results or low specificity of the parasitological test [22, 23]. The finding suggests there is a need to curb misconceptions related to Bf test when diagnosing severe malaria.

In this review, the majority of nurses and clinicians correctly treated severe malaria with both artesunate and antibiotics concurrently to treat co‐infections. These results were consistent with findings from Ghana, Uganda and Malawi, which also showed that the majority of patients with severe malaria were prescribed three doses of artesunate concurrently with antibiotics [7, 24]. In disagreement, a study in Zambia [25] found that a lower percentage of malaria patients were given antibiotics. The difference can be attributed to geographical and political variations among the countries.

The results of this study also revealed that nurses were following a few steps when preparing and administering artesunate. The results are similar to studies in Kenya and Italy, where it was established that the participants knew the preferred route of artesunate administration, which was associated with the availability of artesunate guidelines, and displayed posters on artesunate administration [6, 26]. On the contrary, in Tanzania, the healthcare workers did not know when to discard the preparation if the mixture was not clear or after being used [27]. The findings also exposed that none of the nurses were monitoring the children in the general wards to assess the effectiveness of the treatment. These findings corresponded with studies done in Colombia and Zimbabwe, in which the results also showed that patients with severe malaria and on artesunate were inconsistently monitored [11, 28]. The findings of our study can be attributed to the shortage of staff and equipment, leading to poor outcomes [11, 28]. In this study, nurses and clinicians inadequately provided health education messages to discharge children and their guardians, similar to studies in Malawi and Qatar [23, 25]. In disagreement is a study conducted in Uganda where the children and guardians were advised on how to take drugs, complete treatment at home and use insecticide‐treated nets [22]. The findings of this study can be explained by increased workload, mainly due to a shortage of staff, affecting the length of time spent on a single patient. Lack of information, including malaria prevention measures, could lead to the recurrence of the condition and frequent admissions. This study echoes the need for patient education among paediatric patients. Specifically, health education should also focus on danger signs at home and malaria prevention to prevent the recurrence of the disease.

Finally, it was surprising to note that none of the nurses and clinicians were routinely giving follow‐up dates to discharged patients according to the malaria guidelines. This implied that the discharged under‐five children could not be monitored for any emerging late complications. The results corresponded with what was found in South Asia and Qatar, which noted that none of the severe malaria patients were given follow‐up dates to capture relapses and treat them on time [29]. Contrasting the findings was a study done in Benin, where the results showed that 59.7% of the patients were given follow‐up dates, which helped the providers monitor patients’ delayed complications post‐discharge [25]. The findings of our study suggest that there is a lack of continuity of care. As such, future training, supervision and mentorship should emphasise the impacts of follow‐up protocols to promote the monitoring of patients’ post‐discharge.

### 4.1 Study Limitations

The study is not without limitations. First, the study is cross‐sectional, and although it explains the associated factors, it cannot be used to explain causality. Furthermore, the study was conducted in one district and employed a small sample size; hence, it cannot be generalised to other districts in the country. Second, this study reflected the views and practices of the nurses and clinicians working in paediatric wards only, unlike other departments such as medical and emergency departments, where severe malaria cases are also managed. Additionally, this was a cross‐sectional study that only focused on structures and processes in the management of children with severe malaria and not the outcomes. Furthermore, due to the qualitative nature of the study design, the sample size was not large enough to be generalised. Again, the study could not test hypotheses and measure effect sizes, for example, correlation, chi‐square or the odds ratio. Moreover, the subjective nature of the data collection methods predisposes the study to recall bias. Besides, the study did not employ other qualitative data collection methods, for example, key informant interviews and focus group discussions, which could have helped ensure quality control of the results.

## 5 Conclusion

The study assessed the adherence of healthcare workers to the national guidelines for severe malaria among children in Malawi Hospital Ward. The study found that the nurses and clinicians were not adhering to the national guidelines for malaria, as evidenced by a lack of patient education, a lack of patient follow‐up and a lack of monitoring of children; however, the healthcare providers properly managed children with severe malaria, although the diagnosis was incorrect, possibly due to the use of both MRDT and Bf to diagnose malaria with a slight bias towards MRDT due to mistrust on Bf, which could be attributed to a lack of knowledge about the treatment guidelines. Hence, the study is calling for on‐the‐job training among healthcare workers to familiarise them with the latest guidelines, especially as it relates to children with severe malaria. Further study focusing on the management of severe malaria cases by all nurses and clinicians working in all medical wards is suggested.

## Author Contributions

**Rita Luhanga Soko**: conceptualisation, investigation, writing – original draft, methodology, writing – review and editing, visualisation, formal analysis, project administration. **Chisomo Mulenga**: supervision, methodology, writing – original draft, writing – review and editing, validation. **Thokozani Mzumara**: writing – review and editing, writing – original draft, validation, formal analysis. **Mandayachepa Clifford Nyando**: supervision, writing – review and editing, validation, writing – original draft, methodology, project administration.

## Funding

The authors have nothing to report.

## Conflicts of Interest

The authors declare no conflicts of interest.

## Transparency Statement

Rita Soko affirms that this manuscript is an honest, accurate and transparent account of the study being reported; that no important aspects of the study have been omitted; and that any discrepancies from the study as planned (and, if relevant, registered) have been explained.

## Data Availability

The data are available upon reasonable request through the corresponding author.
